# Enabling factor for cancer hallmark acquisition: Small nucleolar RNA host gene 17

**DOI:** 10.3389/fonc.2022.974939

**Published:** 2022-09-14

**Authors:** Ningzhi Zhang, Yuanyuan Sun, Tuo Wang, Xinyuan Xu, Mengru Cao

**Affiliations:** ^1^ Department of Medical Oncology, Harbin Medical University Cancer Hospital, Harbin, China; ^2^ Medical Affairs Department, Harbin Medical University Cancer Hospital, Harbin, China

**Keywords:** long non-coding RNA, lncRNA small nucleolar host gene 17 (SNHG17), cancer, cancer hallmark, biomarker

## Abstract

The role of long non-coding RNA (lncRNA) in human tumors has gradually received increasing attention in recent years. Particularly, the different functions of lncRNAs in different subcellular localizations have been widely investigated. The upregulation of lncRNA small nucleolar RNA host gene 17 (SNHG17) has been observed in various human tumors. Growing evidence has proved that SNHG17 plays a tumor-promoting role in tumorigenesis and development. This paper describes the molecular mechanisms by which SNHG17 contributes to tumor formation and development. The different functions of SNHG17 in various subcellular localizations are also emphasized: its function in the cytoplasm as a competing endogenous RNA (ceRNA), its action in the nucleus as a transcriptional coactivator, and its function through the polycomb repressive complex 2 (PRC2)-dependent epigenetic modifications that regulate transcriptional processes. Finally, the correlation between SNHG17 and human tumors is summarized. Its potential as a novel prognostic and diagnostic biomarker for cancer is explored especially.

## 1 Introduction

The diversity of cancers increases the complexity of treatment. Hanahan and Weinberg first summarized and classified the functional capabilities acquired by cancer cells that facilitate cell survival, proliferation, and dissemination into six cancer hallmarks in 2000, which include the following: (1) sustaining proliferative signaling, (2) evading growth suppressors, (3) enabling replicative immortality, (4) activating invasion and metastasis, (5) inducing/accessing vasculature, and (6) resisting cell death (1). The following four cancer hallmarks were later added in 2011: deregulating cellular metabolism, avoiding immune destruction, tumor-promoting inflammation, and genome instability/mutation (2). Hanahan (2022) proposed that unlocking phenotypic plasticity, nonmutational epigenetic reprogramming, polymorphic microbiomes, and senescent cells may also be emerging hallmarks of cancer (3). Tumor features in the hallmarks could enable tumors to acquire hallmark capabilities called enabling characteristics, including tumor-promoting inflammation, genome instability/mutation, nonmutational epigenetic reprogramming, and polymorphic microbiomes.

A growing stream of research has focused on the role of non-coding RNAs in tumors. Among them, long non-coding RNAs (lncRNAs) have been found to regulate genomic expression through transcriptional and post-transcriptional levels (4). The function of lncRNA correlates with subcellular localization. LncRNAs can function as competing endogenous RNA (ceRNAs) in the cytoplasm and regulate gene transcription in the nucleus through cis- or trans-regulation (5, 6). This review mainly focuses on the different roles of SNHG17 in various localizations and finds that the effects of SNHG17 emphasized the role of ceRNA in the cytoplasm and regulated transcription in the nucleus as a transcriptional co-activator or through transcriptional repressive chromatin modifications. The vital role of epigenetic modifications has been gaining attention in recent years. Thus, the role of the epigenetic modification function of SNHG17 in promoting tumorigenesis and progression is also discussed.

SNHG is the small nucleolar host gene and belongs to lncRNA. Several SNHG family members, such as SNHG1 (7, 8), SNHG20 (9), SNHG3 (10, 11), SNHG5 (12, 13), and SNHG16 (14), are recently closely associated with tumors. Meanwhile, as the homolog of SNHG, snoRNA has a connection with SNHG. snoRNA plays a role in ribosomal RNA modification, stress response, and tumor development. However, but the relationship between snoRNA and SNHG is unclear. Previous literature describes that SNHG17 in prostate cancer increases the expression of its homolog snoRA71B through a positive feedback loop, which promotes tumor progression (15). The regulatory relationship between SNHG and snoRNA is also investigated in a new insight.

This paper discusses the correlation between SNHG17 and tumors by describing the molecular mechanisms by which SNHG17 contributes to the formation of cellular hallmark capabilities and the enabling characteristics. The paper also focuses on its different functions in various subcellular localizations and finally discusses the potential of SNHG17 as a new prognostic and diagnostic biomarker for cancer.

## 2 Materials and methods

### 2.1 Raw data

The data samples of the differentially expressed genes of LUAD after SNHG17 knockdown were downloaded from GSE131543. The immune-related genes were acquired from the ImmPort database.

### 2.2 GO analyze

GO analyze was performed using Metascape (http://metascape.org/). Terms with p-value cutoff of 0.01, min overlap of 3, and min enrichment of 1.5 were considered. The top 20 enriched terms are displayed in **Figure 4B**.

### 2.3 Immune and stromal infiltration analysis

ssGSEA was applied to explore the infiltration degrees of immune cell types in LUAD of the TCGA database using the GSVAR package in R (version 1.34.0). The estimated package was used to generate ImmuneScore, StromalScore, and ESTIMATEScore. R language version 3.6.3 loaded with ggplot2 package (version 3.3.3) was used to demonstrate the correlation between SNHG17 and PD-L1. All the correlation between SNHG17 and others were studied using Spearman correlation analysis.

## 3 Results

### 3.1 Association of SNHG17 in the acquisition of hallmark capabilities

#### 3.1.1 Role of SNHG17 in sustaining proliferative signaling

**Figure 1:**
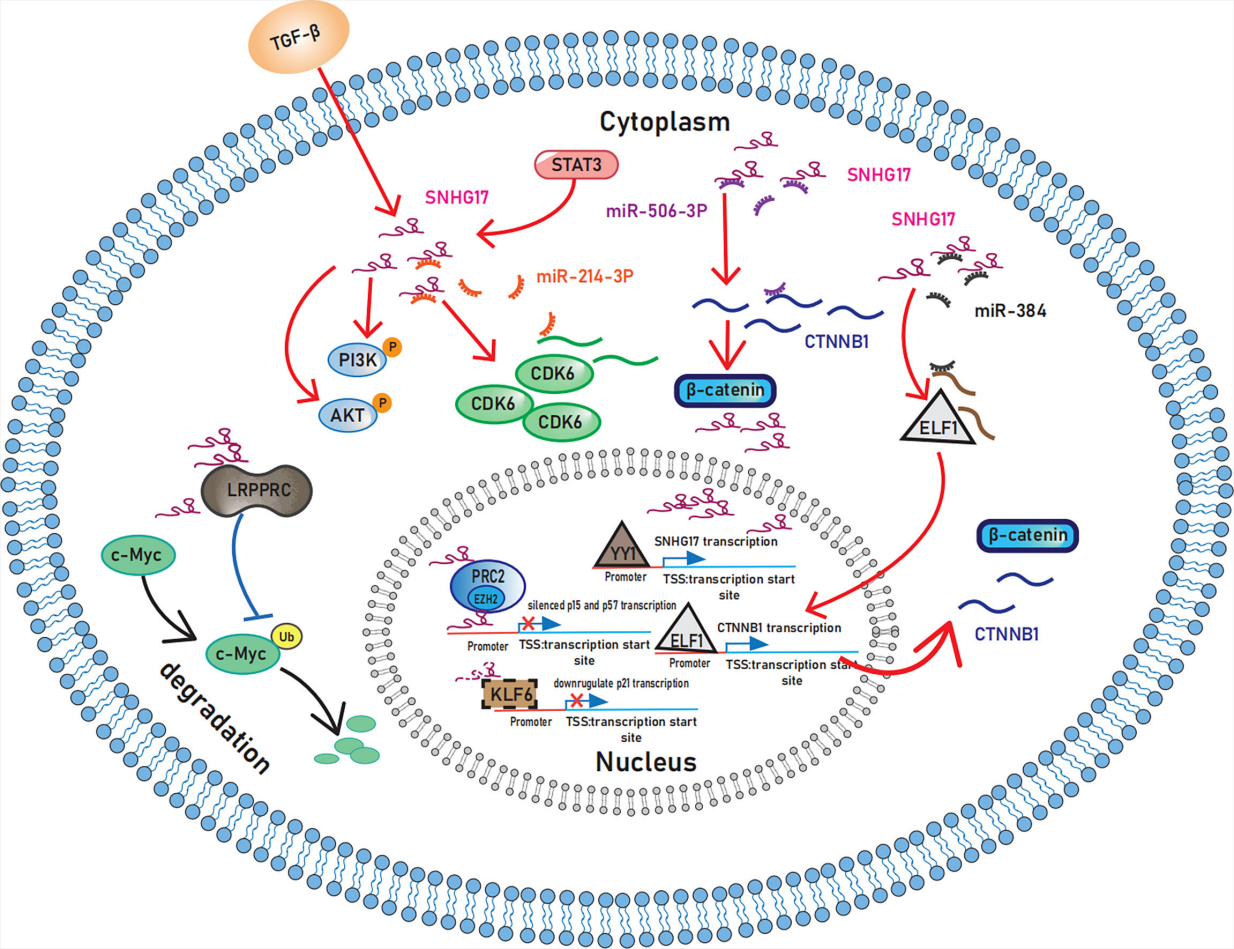
Schematic representation of the mechanisms by which SNHG17 plays in sustaining proliferative signaling. In the cytoplasm, SNHG17 acts as the ceRNA for miR-506-3 and miR-384 to activate the Wnt/β-catenin pathway and enhance the expression of CDK6 by targeting miR-214-3p. Moreover, STAT3 and TGF-β activate the PI3K/AKT signaling pathway by increasing SNHG17 levels. By interacting with LRPPRC, SNHG17 can also reduce c-Myc ubiquitination. In nucleus, SNHG17 targets p57 and p15 by binding to EZH2 and PRC2. p21 may also exist downstream of SNHG17 considering the regulatory relationship between KLF6 and SNHG17. And transcription factor YY1 upregulate the transcription of SNHG17. SNHG17, lncRNA small nucleolar RNA host gene 17; CDK6, Cyclin-dependent kinase 6; STAT3, Signal transducer and activator of transcription 3; TGF-β, Transforming growth factor β; PI3K, Phosphoinositide-3 kinase; AKT, Protein kinase B; LRPPRC, Leucine-rich pentatricopeptide repeat-containing protein; EZH2, enhancers on zeste homolog 2; PRC2, Polycomb repressive complex 2; KLF6: Krupple-like factor 6; YY1, Yin-yang 1.

SNHG17 is associated with increased cell proliferation capacity in various types of cancers. The mechanisms that promote the formation of this phenotype include the activation of cyclin-dependent kinases (CDKs), phosphoinositide-3 kinase/protein kinase B (PI3K/Akt) and Wnt/β-catenin signaling pathway, and the inhibition of cyclin-dependent kinase inhibitor (CKI) (**Figure 1**).

##### 3.1.1.1 SNHG17 regulates CKI, CDK, and c-Myc

Ma Z et al. (16 *)*, first identified the relevance between SNHG17 and tumor cell proliferation in colorectal cancer, and revealed that SNHG17 epigenetically targets p57 by binding to enhancers on zeste homolog 2 (EZH2). As a well-known CKI, p57 plays a vital role in regulating the cell cycle. Briefly, the cell cycle regulatory machinery comprises three major types of proteins: cyclin, CDK, and CKI. The binding of cyclin with CDK promotes cell cycle progression, and CKI hinders this effect by inhibiting the cyclin-CDK complex. The tumor suppressor-like properties of CKI have been reported in recent years (17), and lncRNAs can promote cancer cell proliferation and migration by decreasing CKI expression (18). SNHG17 acts as a suppressor of multiple CKIs in tumors; in addition to p57, SNHG17 can also inhibit p15 and p16, and this CKI inhibitory property contributes to increasing cell proliferation capacity (19, 20). Moreover, RNA sequencing predicts that SNHG17 can interact with the transcription factor krupple-like factor 6 (KLF6) (21). p21 may also exist downstream of SNHG17 considering the regulatory relationship between KLF6 and p21 (22). Furthermore, SNHG17 can enhance the expression of CDK4 and CDK6, promoting cell cycle progression (20, 23).

The role of SNHG17 in the cell cycle also involves the ubiquitin–proteasome system (UPS). As a considerably method for intracellular protein degradation, the UPS is one of the essential mechanisms controlling the levels of MYC protein (24). A recent study in hepatocellular carcinoma found that SNHG17 can reduce c-Myc ubiquitination, increase c-Myc levels, and promote G1/S phase transition (25). SNHG17 can play multiple roles through UPS. For example, in colorectal cancer, SNHG17 competes with the E3 ligase Trim23 to bind Pescadillo (PES1), protecting PES1 from degradation (26). In non-tumor diseases, SNHG17 reduces MST ubiquitination and degradation and regulates the apoptosis of podocytes and Parkin-dependent mitophagy in diabetic nephropathy (27). However, the relationship between SNHG17 and UPS currently needs rigorous experimental data support, and this area of research requires further exploration.

##### 3.1.1.2 SNHG17 regulates PI3K/AKT signaling pathway

PI3K/AKT signaling, one of the critical intracellular pathways, is also involved in the mechanism of how SNHG17 enables tumor cells to obtain the capacity of sustained proliferation. STAT3 and transforming growth factor-β (TGF-β) are located upstream of SNHG17, which contribute to activate the PI3K/AKT signaling pathway by increasing SNHG17 levels (28, 29). In addition, SNHG17 may indirectly activate the PI3K/AKT signaling pathway through its regulatory effects on NETO2 and PES1 (26, 30–32).

##### 3.1.1.3 SNHG17 regulates Wnt/β-catenin signaling pathway

SNHG17 acts as the ceRNA for miR-506-3p and miR-384 in the cytoplasm to exert pro-proliferative effects through the Wnt/β-catenin pathway (33, 34). For the specific mechanism, SNHG17 affects the *CTNNB1* gene, which encodes β-catenin by targeting miR-506-3p (33) and miR-384 to act on the transcription factor ELF1 (34) to promote the transcription of the *CTNNB1* gene. Moreover, transcription factor yin-yang 1 (YY1) facilitates this upstream regulation of SNHG17 (33). Intriguingly, Wnt ligand secretion mediator (WLS) and stanniocalcin 2 (STC2) are involved in the pro-proliferative effects of SNHG17 as downstream targets (35, 36), which are both closely related to the Wnt/β-catenin signaling pathway (37, 38). However, additional research needs to verify whether SNHG17 can activate the Wnt/β-catenin pathway and thus promote tumor progression through WLS and STC2.

#### 3.1.2 Role of SNHG17 in forming death-resistant phenotype

The forms of cell death include accidental cell death (ACD) and regulatory cell death (RCD). Apoptosis has been widely investigated as the first identified regulatory cell death in tumors; some non-apoptotic regulatory cell deaths, such as autophagic cell death, pyroptosis, and ferroptosis, are also currently gaining attention. Cancer cells can resist cell death through multiple pathways, which is also one of the hallmark capabilities. Interestingly, the upregulation of SNHG17 is associated with increased drug resistance in astrocytoma and prostate cancer (39, 40). Furthermore, several studies have shown the formation mechanisms of the death-resistant phenotype. Herein, several reported mechanisms are introduced, and examples for known modes of action are provided.

##### 3.1.2.1 SNHG17 regulates apoptosis-related proteins

Multiple innate tumor suppressive mechanisms exist in mammals to ensure that cells have normal levels of proliferation, and these mechanisms are activated when cells show aberrant proliferation, leading to apoptosis or senescence (41). Thus, resistance to apoptosis is a barrier that must be breached for tumor formation. The anti-apoptotic effect of SNHG17 on tumor cells has been currently observed in a variety of tumors, including cervical cancer, pancreatic cancer, astrocytoma, hepatocellular carcinoma, oral squamous cell carcinoma, prostate cancer, ovarian cancer, gastric cancer, melanoma, and colorectal cancer (15, 16, 19, 23, 28, 39, 42–45). This anti-apoptotic mechanism was associated with increased anti-apoptotic protein Bcl-2 and the decreased activity of the pro-apoptotic proteins caspase3, caspase8, caspase9, and Bax (33, 35, 36, 40, 46, 47). In addition, RNA-seq analysis performed in non-small cell lung cancer revealed that the genes of the pro-apoptotic proteins BIK and XIAP-associated factor 1 (XAF1) are the downstream targets of SNHG17 (48).

In addition, IGF binding protein 3 (IGFBP3) may act as a bridge linking SNHG17 to p53-dependent apoptosis in colorectal cancer (21). IGFBP3 promotes colorectal cancer progression through p53-dependent apoptosis (49). Notably, Parkin protein was recently found to be regulated by SNHG17 in non-tumor diseases (27). Parkin is a ubiquitin-protein ligase (E3), which can be involved in the regulation of apoptosis by ubiquitinating various apoptosis-related proteins. Notably, studies on whether SNHG17 can regulate apoptosis through Parkin protein in tumors, which may become a director in future SNHG17 investigations, are lacking.

##### 3.1.2.2 SNHG17 regulates autophagy-related protein

Autophagy plays a role in the maintenance of normal cellular homeostasis-like apoptosis. Interestingly, as with TGF-β, cellular autophagy plays a dual role in tumor progression, inhibiting early tumor formation and promoting late tumor progression (50–52). Increased mitochondrial autophagy in tumors leads to high chemo- and radiotherapy resistance by increasing metabolic plasticity in cancer cells (53–55). This section further described cellular metabolism deregulation below. Overall, mitochondrial autophagy has a vital role in tumorigenesis and development.

SNHG17 was screened as an effective autophagy-related lncRNA signature closely associated with prognosis in ovarian cancer and renal clear cell carcinoma (56, 57). Intriguingly, SNHG17 has been shown to reduce Parkin-dependent mitochondrial autophagy by regulating Parkin proteins in non-tumor diseases (27). Parkin is known to act as a mitophagy initiator, and Parkin-dependent mitochondrial autophagy is downregulated in various tumors and has been suggested to belong to a tumor suppressor mechanism (58). Studies on whether SNHG17 can regulate Parkin-dependent mitochondrial autophagy and affect tumor progression, which requires further experimental verification in the future, are still unavailable.

##### 3.1.2.3 SNHG17 is associated with ferroptosis

Ferroptosis is an iron-dependent oxidative cell death (59), and this cell death pathway could be one of the directions for tumor therapy (60). SNHG17 is involved in tumor progression as a ferroptosis-associated lncRNA. Risk assessment and diagnostic models constructed with ferroptosis-associated lncRNAs, such as SNHG17, have shown excellent prognostic and diagnostic values in renal cancer (61). However, further studies are still needed to elucidate the mechanistic role of SNHG17 in ferroptosis.

#### 3.1.3 Role of SNHG17 in accelerating EMT process

Concerning the studies on the relationship between SNHG17 and tumors, the most significant relationship is the promotion of tumor proliferation, invasion, and metastasis. Several studies have shown that the high-level expression of SNHG7 correlates with lymph node metastasis, distant metastasis, and tumor invasion depth in various tumors (19, 29, 42, 44, 46). Spreading to distant organs is recognized to be the most prominent hallmark of cancer cells. A primary biological process that sustains invasion and 1metastasis is the epithelial–mesenchymal transition (EMT), wherein cells gain the mesenchymal phenotypes, such as high migration and invasion and the capability to degrade extracellular matrix.

SNHG17 can have different functions in various subcellular localizations. The best-known capability of SNHG17 lies in its function as ceRNA in the cytoplasm. In esophageal squamous and hepatocellular carcinomas, SNHG17 can promote EMT by targeting miR-338-3p/SOX4 and miR-3180-3p/regulatory factor X-box 1 (RFX1) axes, respectively (62, 63).

**Figure 2:**
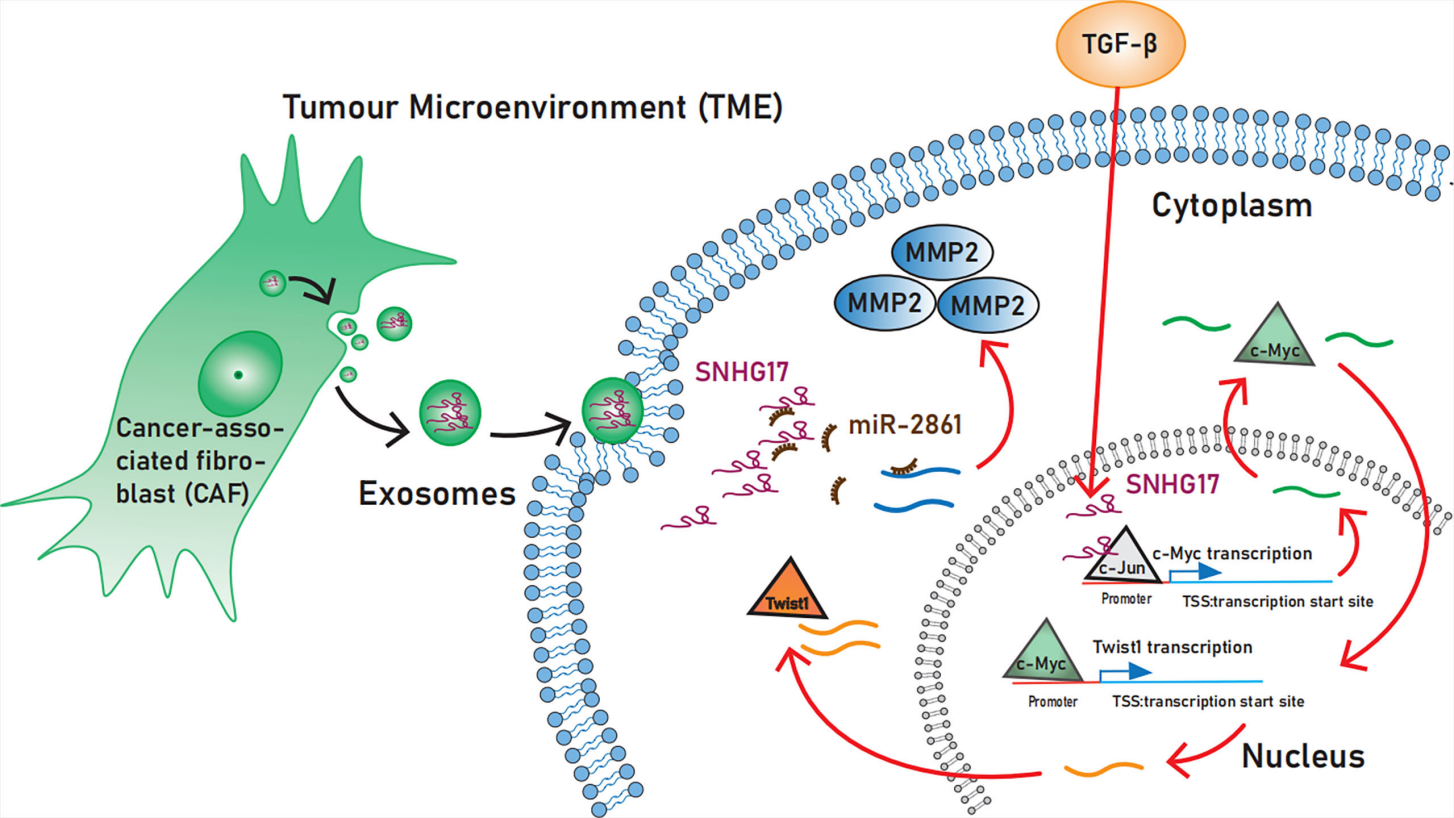
The role SNHG17 plays in activating invasion and metastasis. SNHG17 acts as a transcriptional co-activator by recruiting the transcription factor c-Jun to the c-Myc promoter region, and promotes the expression of the EMT-associated transcription factor Twist1 *via* c-Myc. In the Extracellular, SNHG17 can be secreted by tumor-associated fibroblasts and target MMP2 in the form of exosomes. EMT, Epithelial-mesenchymal transition; MMP2, Matrix metalloproteinases 2.

The presence of positive feedback regulatory loop SNHG17/miR-339-5p/STAT5A/SNHG17 and SNORA71B in prostate cancer can promote cellular EMT and thus facilitate cancer progress (15). Similarly, in castration-resistant prostate cancer, SNHG17 regulates CD51 and thus promotes tumor EMT *via* sponge miR-144 (64) (**Figure 2**).

##### 3.1.3.1 SNHG17 regulates EMT process *via* TGF-β, Twist 1 and c-Myc

TGF-β is widely known to play an important role in cancer. In the early stages, TGF-β exerts an oncogenic effect by inhibiting the cell cycle process; in the late stages, it can induce invasion and metastasis and promote EMT (65).

TGF-β-induced SNHG17 hyperactivation promotes EMT. Unlike in the cytoplasm, lncRNAs can regulate the transcriptional process of some oncogenes by binding to transcription factors while in the nucleus (66). Shen S et al. (29 *)*, recently demonstrated that SNHG17 is involved in TGF-β1-mediated EMT in esophageal squamous cell carcinoma. Mechanistically, SNHG17 acts in the nucleus by recruiting the transcription factor c-Jun to the c-Myc promoter region, which increases the transcriptional activity of the c-Myc promoter. Furthermore, SNHG17 promotes the expression of the EMT-associated transcription factor Twist1 *via* c-Myc (29).

##### 3.1.3.2 SNHG17 regulates EMT process *via* matrix metalloproteinases

Matrix metalloproteinases (MMPs) belong to the family of extracellular proteases and promote tumor progression through tumor microenvironment (TME) regulation. MMPs are not only known as a marker of EMT but also induce EMT. Exosomes can act as tools for cancer cells to regulate the TME and promote proliferation and invasion. As the lncRNA released from tumor-derived exosome, SNHG17 is crucial in promoting tumor migration by increasing MMP2 levels through sponge miR-2861 (47).

##### 3.1.3.3 SNHG17 regulates EMT process *via* Wnt/β-catenin signaling pathway

The role of the Wnt/β-catenin signaling pathway in EMT is widely recognized. However, the relationship between SNHG17 and Wnt/β-catenin signaling pathway is yet to be investigated. For reference, STC2, a downstream target of miR-361-3p, was shown to promote tumor invasion and migration *via* the Wnt/β-catenin signaling pathway *in vivo* experiments (38), while SNHG17 acts as a ceRNA for miR-361-3p (36).

#### 3.1.4 Role of SNHG17 in increasing tumor angiogenesis

The pro-angiogenic effect of SNHG17 can be achieved by sponging miR-23a-3p and thus regulating the chemokine CXCL12 (67). In addition, SNHG17 can target miR-942 to regulate vascular endothelial growth factor (VEGF) expression (43), but the exact mechanism needs further exploration. H2AX, which can be regulated by SNHG17, also plays an essential role in tumor angiogenesis (46, 68). Intriguingly, CDK6 can promote tumor angiogenesis through kinase-independent function; the transcriptional regulator activity of CDK6 provides new ideas for the functional exploration of SNHG17 (23, 69).

#### 3.1.5 Role of SNHG17 in deregulating cellular metabolism

Although no studies in tumors have confirmed the direct effect of SNHG17 on regulating cellular metabolism, a regulatory relationship may exist between SNHG17 and deregulating cellular metabolism according to the mechanism exploration of SNHG17 in some studies.

##### 3.1.5.1 SNHG17 regulates abnormal glucose metabolism

Considering the non-negligible role of cellular metabolism in cancer development, deregulating cellular metabolism was included in the cancer hallmarks as an emerging hallmark by Hanahan and Weinberg (2, 70). Otto Warburg first identified the Warburg effect regarding cancer cells having different energy metabolism from normal cells even under the aerobic environment; cancer cells still prefer to obtain energy through the glycolytic pathway (71, 72). This metabolic mechanism is seemingly counterintuitive because glycolysis is much less efficient than mitochondrial oxidative phosphorylation. However, this particular metabolic is conducive to rapid cell proliferation (73), and metabolic plasticity can act as a survival mechanism of tumors under cancer treatment (53).

Mitochondrial autophagy proves tumors with metabolic plasticity by degrading cellular structures and recycling metabolites in response to environmental stress (54, 55). As described in the resisting cell death section above, SNHG17 reduces mitochondrial autophagy by downregulating Parkin in non-tumor diseases (27), and Parkin plays a role in glucose metabolism and the Warburg effect as a p53 target gene (74). Furthermore, as the E3 ubiquitin ligase, Parkin can promote hypoxia inducible factor-1α (HIF-1α) degradation through ubiquitination and proteasomal degradation (75). HIF-1α is known to act as a hypoxia-inducible transcription factor and can promote the Warburg effect by regulating cellular metabolism (76).

Several transcription factors are involved in establishing the Warburg effect. One factor, namely c-Myc, can confer metabolic advantages to tumor cells by regulating the expression of multiple genes (77, 78). Interestingly, SNHG17 has been demonstrated in several studies to increase c-Myc levels by promoting transcription with inhibition of ubiquitination and degradation (25, 26, 29). c-Myc may also serve as a bridge for SNHG17 to establish the Warburg effect in cancer cells.

##### 3.1.5.2 SNHG17 modulates lipid metabolism

Previous interest in tumor metabolic abnormalities has focused on glucose metabolism. However, recent studies have shown that abnormalities in lipid metabolism are also present in tumors. Reprogramming of lipid metabolism can contribute to tumorigenesis and drug resistance (79–81). Low-density lipoprotein cholesterol (LDL-C) levels are positively associated with increased cancer risk (82). Relevant studies on tumors are lacking. However, SNHG17 was found to be negatively associated with high-density lipoprotein cholesterol (HDL-C) levels in a study on diabetes and may be involved in the formation of type 2 diabetes (83). This study demonstrates a regulatory relationship between SNHG17 and HDL-C and the possible involvement of SNHG17 in tumor progression through lipid metabolism. Moreover, lipid metabolism may become a new direction to explore the tumor-promoting mechanism of SNHG17.

#### 3.1.6 Role of SNHG17 in regulating telomerase activity

The contribution of SNHG17 to achieving replicative immortality has not been fully investigated. However, the telomerase regulatory activity of the SNHG17-associated protein pescadillo (PES1) deserves consideration. Telomeres, which protect the ends of the chromosome, are closely associated with the capability of tumor cells to proliferate indefinitely. Consequently, telomerase, specifically expressed in most tumors, plays an important role and prevents telomere shortening. Telomerase is an attractive target for tumor therapy. Its catalytic core includes telomerase reverse transcriptase and telomerase RNA. SNHG17-related protein PES1 can promote tumor progression in multiple ways. As a key component of telomerase composition, PES1 promotes telomerase assembly by facilitating the direct interaction between telomerase reverse transcriptase and telomerase RNA. The increased expression of PES1 can also lead to enhanced telomerase activity and affect telomere length maintenance (26, 84).

#### 3.1.7 Role of SNHG17 in avoiding immune destruction

Hanahan and Weinberg (2011) introduced immune destruction avoidance as another hallmark capability (2). The extensive crosstalk between the tumor microenvironment (TME) and the tumor promotes the capability of avoiding immune destruction.

Cancer-derived exosomes have been of broad interest as the tumor TME component, which plays a vital role in tumor occurrence and development (85). They have recently been proposed to have the potential to become emerging enabling characteristics (86). SNHG17 can be secreted by tumor-associated fibroblasts in the form of exosomes and targets MMP2 (47). Matrix metalloproteinases 2 (MMP2) promotes immunosuppressive TME formation by reducing anti-tumor-associated immune cells (CD4+ and CD8+ T cells, NK cells, and CD103+ DCs) and increasing M2-like macrophages (87).

#### 3.1.8 Role of SNHG17 in unlocking phenotypic plasticity

According to “Hallmarks of Cancer: New Dimensions” reported by Hanahan in 2022, unlocking phenotypic plasticity may be included in the hallmark capabilities (3). Unlocking phenotypic plasticity is mainly manifested by blocking normal cell differentiation in developmental lineages. Notably, as cell development-related transcription factors involved in this process, SOX2, SOX4, and homeobox A1 (HOXA1) can be regulated by SNHG17 (3, 30, 39, 62, 88).

#### 3.1.9 Role of SNHG17 in regulating senescent cells

Many studies have shown that senescent cells promote tumor proliferation, migration, and other malignant behaviors through senescence-associated secretory phenotype (89–93). Therefore, senescent cells are also classified as potential emerging tumor markers (3). Genomic instability is one of the significant causes of senescence, and the relationship between SNHG17 and genomic instability will be described in the genome instability/mutation section; meanwhile, the senescence-associated secretory phenotype regulator NF-κB is involved in maintaining high levels of SNHG17 expression (94).

### 3.2 Association of SNHG17 in the development of enabling characteristics

#### 3.2.1 Role of SNHG17 in induces genome instability/mutation

The formation of most cancer hallmarks is associated with genome dysfunction. Genome maintenance systems provide numerous contributions to maintain the stability of the genome.

The impairment of caretaker mechanisms, such as DNA repair defect, aberrant cell cycle regulation, and abnormal telomere DNA maintenance mechanisms, can lead to genome instability (95). As described in the sustaining proliferative signaling section, SNHG17 has been found to inhibit cell cycle protein-dependent kinase inhibitor (CKI) p15, 16, 57, and to increase (16, 19, 20, 96) cell cycle protein-dependent kinase CDK4 and CDK6 expression (20, 23) to involve in aberrant cell cycle regulation.

##### 3.2.1.1 SNHG17 affects the selection of DNA damage repair pathway

**Figure 3:**
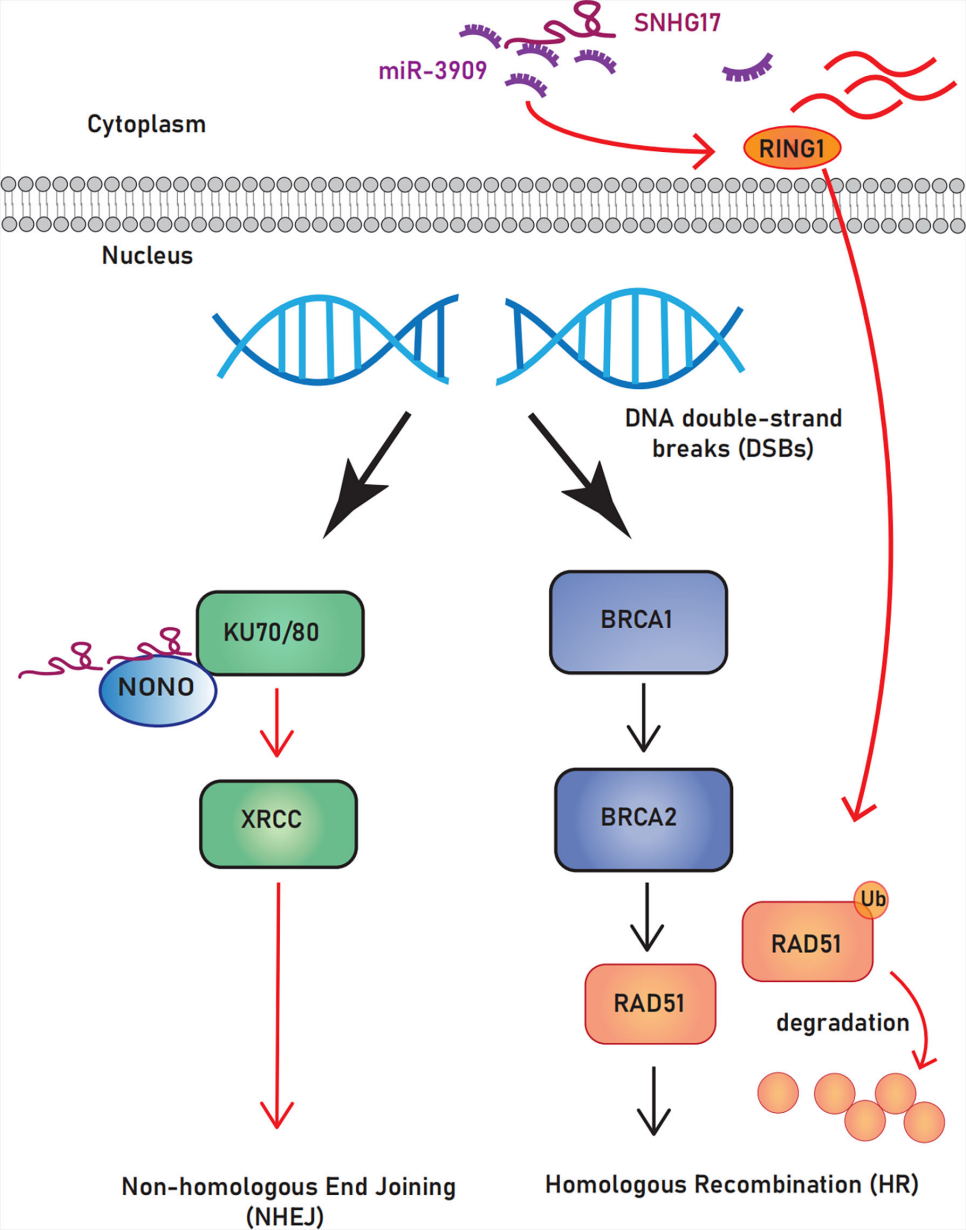
The role of SNHG17 plays in DNA repair.

Most cancers are characterized by genome instability/mutation, giving cells a selective growth advantage (21, 97). Multiple repair pathways are available after DNA damage; compared with homologous recombination, non-homologous end joining leads to genomic instability and is conducive to tumor development because it cannot guarantee repair accuracy (98). Notably, SNHG17 can promote the non-homologous end joining pathway by targeting non-POU domain-containing octamer-binding protein and acts as a ceRNA to downregulate the expression of the central homologous recombination protein Rad51 (94), which predisposes DNA double-strand breaks to select the non-homologous end joining repair pathway. The role of SNHG17 in DNA repair makes it a potential biomarker for cancer diagnosis and treatment (**Figure 3**).

##### 3.2.1.2 SNHG17 affects telomerase activity

The relationship between SNHG17 and telomerase lacks support from additional rigorous experimental data. For reference, SNHG17 can regulate the ubiquitinate level of PES1 (26), and PES1 can interact with telomerase reverse transcriptase to regulate telomerase activity (84).

#### 3.2.2 Role of SNHG17 in tumor-promoting inflammation

Appropriate inflammation has cancer-suppressing effects on cancers as part of innate immunity, but chronic inflammatory cell infiltration leads to an increased risk of tumor formation. The inflammation that precedes tumor development promotes tumorigenesis by causing genomic instability, recruiting growth factors, and angiogenesis. By contrast, tumor-associated inflammation can lead to immunosuppression addition (99–101).

SNHG17 is a new link between inflammation and cancer. In a study on h. pylori-induced gastric carcinogenesis, SNHG17 was upregulated by the inflammation-associated transcription factor NF-κB; as described in the genome stability/mutation section, SNHG17 consequently affected the selection of the DNA Damage Repair pathway, thereby promoting gastric carcinogenesis (94).

**Figure 4:**
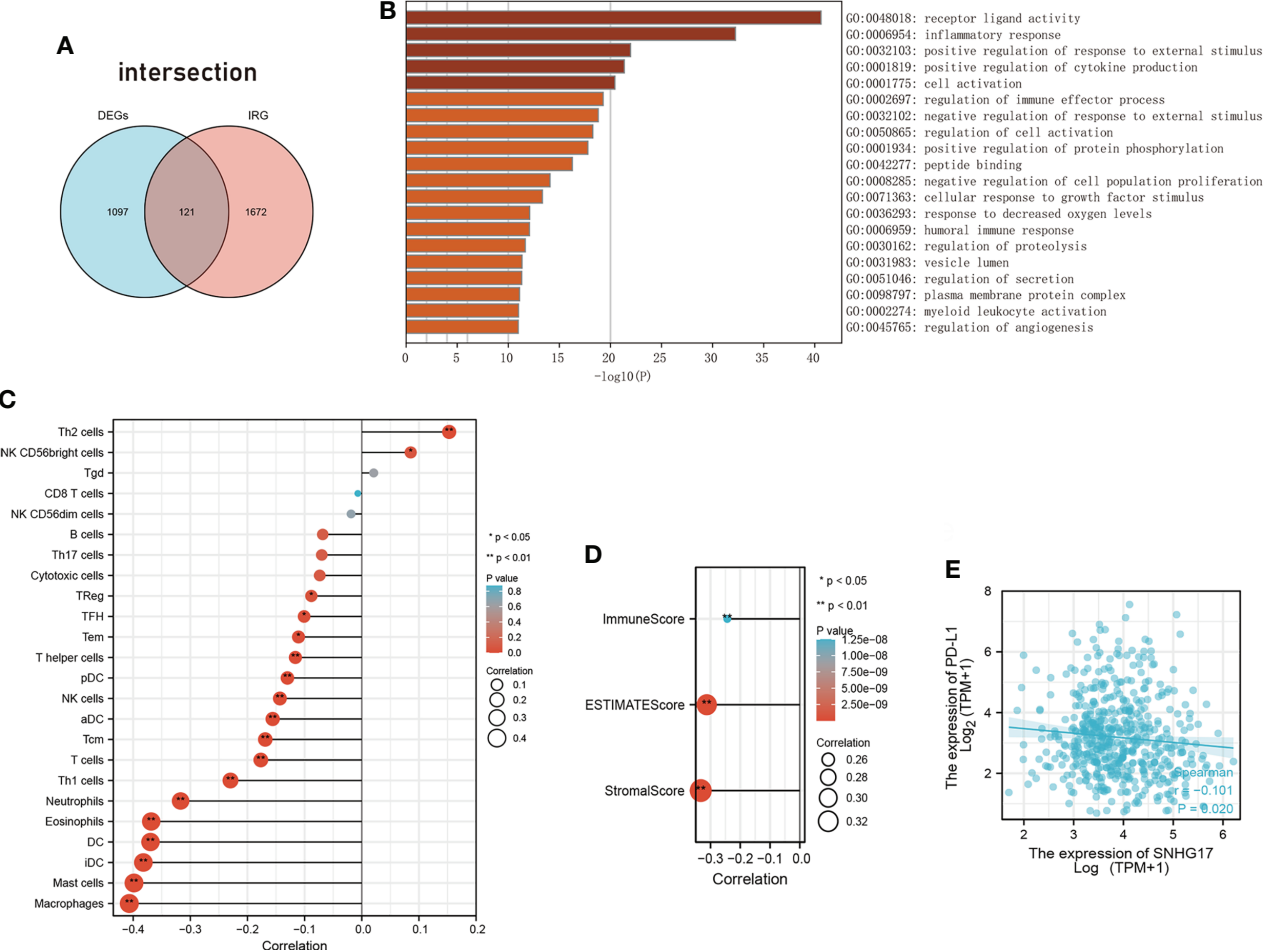
The relationship between SNHG17 and immune. **(A)** Venn diagram visualizing the overleaping genes between DEGs and IRGs. **(B)** Summary of enrichment analysis across input the intersected genes of DEGs with IRGs. **(C)** The correlation between SNHG17 and immune cell infiltration, respectively. **(D)** Correlation analysis between SNHG17 and immune-related scores (ESTIMATEScore, ImmuneScore, StromalScore). **(E)** Correlation analysis between SNHG17 and PD-L1 expression. DEGs, Differentially expressed genes; IRGs, Immunity-related genes; PD-L1, Programmed death ligand 1.

Considering the extensive search for inflammation and immunity in tumor formation and progression, the correlation between SNHG17 and inflammation and immunity was further explored. A set of differentially expressed genes was used after knocking down SNHG17 in lung adenocarcinoma (LUAD) and intersected them with immune-related genes downloaded from the ImmPort database (**Figure 4A**) (**Supplementary Data Sheet 1**). Gene ontology (GO) enrichment analysis indicated that these intersected genes mainly focused on receptor ligand activity, inflammatory response, positive regulation of response to external stimulus, positive regulation of cytokine production, cell activation, and the regulation of immune effector process (**Figure 4B**) (**Supplementary Data Sheet 2**). SNHG17 was almost mapped to the inflammatory response in the innate immune response. The relationship between SNHG17 expression levels and immune infiltration was then explored. **Figure 4** shows the following: SNHG17 had a positive correlation with Th2 and NK CD56 bright cells and a negative correlation with Macrophages, DCs, Neutrophils, Th1 cells, T cells; it was negatively correlated to ESTIMATEScore, ImmuneScore, StromalScore, and PD-L1 (**Figures 4C-E**) (**Supplementary Data Sheet 3–5**).

In addition, the SNHG17-associated protein MMP2 is involved in the formation of the inflammatory TME through TLR2 and TLR4 (47, 87).

#### 3.2.3 New dimensions: Nonmutational epigenetic reprogramming

Nonmutational epigenetic reprogramming is a possible enabling characteristic newly proposed by Hanahan (3). Epigenetic modifications are prevalent in many cancers and facilitate cancer hallmark formation. SNHG17 is involved in nonmutational epigenetic reprogramming mainly by interacting with the chromatin-associated methyltransferase polycomb repressive complex dependent 2 (16, 19) and blocking ubiquitination and degradation (25, 26).

In addition, as the spliced transcripts of the same primary transcript, SNHG and snoRNA theoretically have the same promoter. A recent study demonstrated a positive feedback loop between SNHG17 and its homolog snoRA71B, promoting tumor progression. Specifically, SNHG17 can promote transcription factor STAT5A expression by targeting miR-339-5p, thereby increasing SNHG17 and cognate snoRA71B levels (15). This finding further confirms the link between SNHG and snoRNA. SnoRNA is known to play an oncogenic role in a variety of tumors (102), and its broadest function is the epigenetic modification of rRNA at the post-transcriptional level through 2’-O-methylation and pseudouridylation. The high relevance to the response to immune therapy and the broad function beyond the ribosome of snoRNA is recently gaining attention. The relationship between SNHG and snoRNA is currently an open question. However, SNHG promotes the expression of its cognate snoRNA through the same promoter positive feedback, thus exerting oncogenic effects through snoRNA. The feedback may become a worthy direction for the subsequent study of SNHG and snoRNA.

#### 3.2.4 Investigating feature: Cancer-derived exosomes

Cancer-derived exosomes can be observed in most cancers, promoting the formation of multiple hallmarks, which is in line with Hanahan’s definition of enabling features (3). Moreover, Kok VC et al. (86 *)*, recently raised the possibility of cancer-derived exosomes as an emerging enabling characteristic. Interestingly, SNHG17 can be released by cancer-associated fibroblast as exosomal lncRNA in osteosarcoma to promote tumor proliferation, migration, and apoptosis resistance (47).

## 4 Conclusion

SNHG17 has been found to be involved in forming cancer hallmarks in numerous ways. SNHG17 acts as ceRNA in the cytoplasm. Meanwhile, SNHG17 in the nucleus can act as a transcriptional co-activator or repress chromatin modifications transcriptionally to increase the malignant progression. These different functions suggest that SNHG17 plays a diverse role in tumorigenesis and progression. Thus, targeting SNHG17 may become a promising strategy for tumor therapy. In addition, SNHG17 can be an independent prognostic factor in hepatocellular carcinoma, renal cell carcinoma, colorectal cancer, gastric cancer, and melanoma (26, 28, 44, 46, 103) and a diagnostic predictor in cervical and gastric cancers (19, 42) (**Table 1**). Therefore, SNHG17 is an emerging biomarker for cancer diagnosis and prognosis.

**Table 1:** Expression and clinical significance of SNHG17 in human cancers.

Cancer Types	SNHG17 Expression	Clinical Characteristics				References
		Kaplan-Meier Survival Analysis	Independent Prognosis Predictor	Clinicopathological Characteristics	Diagnostic Value	
Gastric Cancer	Upregulated	poorer OS^[94,103]^,PFS^[103]^	yes^[103]^	TNM stage^[18,19,94,103]^, lymph node metastasis^[18,19,103]^, younger age^[18]^, invasion depth^[18]^, lymphovascular invasion^[19]^, distant metastasis^[103]^, H.pylori infection^[94]^	plasma SNHG17(AUC 0.748)^[18]^	(18, 19, 94, 103)
Hepatocellular Carcinoma	Upregulated	poorer OS^[24,43]^,DFS^[43]^, RFS^[24]^	yes^[43]^	Tumor size^[43,63]^, poor differentiation^[43]^, vascular invasion^[43]^, TNM stage^[63]^, Edmonson-Steiner grades^[63]^	–	(24, 43, 63)
Prostate Cancer	Upregulated	poorer OS^[39]^, PFS^[45]^	–	Histological grade^[39]^, tumor stage^[39]^, metastasis^[39]^	–	(39, 45)
Esophageal Squamous Cell Carcinoma	Upregulated	poorer OS	yes	TNM stage, grade, depth of invasion, tumor differentiation, lymph node metastasis, mortality	–	(28)
Renal Cell Carcinoma	Upregulated	poorer OS, RFS	yes	Tumor size, lymph node invasion, distant metastasis, relapse status	–	(46)
Colorectal Cancer	Upregulated	poorer OS ,DFS	yes	Tumor stage	–	(25)
Lung Adenocarcinoma	Upregulated	poorer OS	–	higher in stages III and IV	–	(29)
Cervical Cancer	Upregulated	–	–	FIGO stage, lymph node metastasis, tumor diameter	AUC 0.863	(41)
Ovarian Cancer	Upregulated	poorer OS	–	FIGO stage, histological grade, tumor size	–	(22)
Breast Cancer	Upregulated	poorer OS	–	TNM stages (III–IV stages), lymph node metastasis	–	(39)
Tongue Squamous Cell Carcinoma	Upregulated	poorer OS	–	Tumor size, TNM stage, lymph node metastasis	–	(23)
Glioma	Upregulated	poorer OS	–	–	–	(27)
Osteosarcoma	Upregulated	poorer OS	–	–	–	(47)
Melanoma	Upregulated	poorer OS	yes	Tumor stage, lymph node metastasis, tumor stage	–	(27)

OS: shorter overall survival; DFS: disease-free survival; RFS, recurrence-free survival; AUC, area under the ROC curve; TNM, tumor node metastasis; PFS, progression-free survival; FIGO, international federation of gynecology and obstetrics.

**Figure 5:**
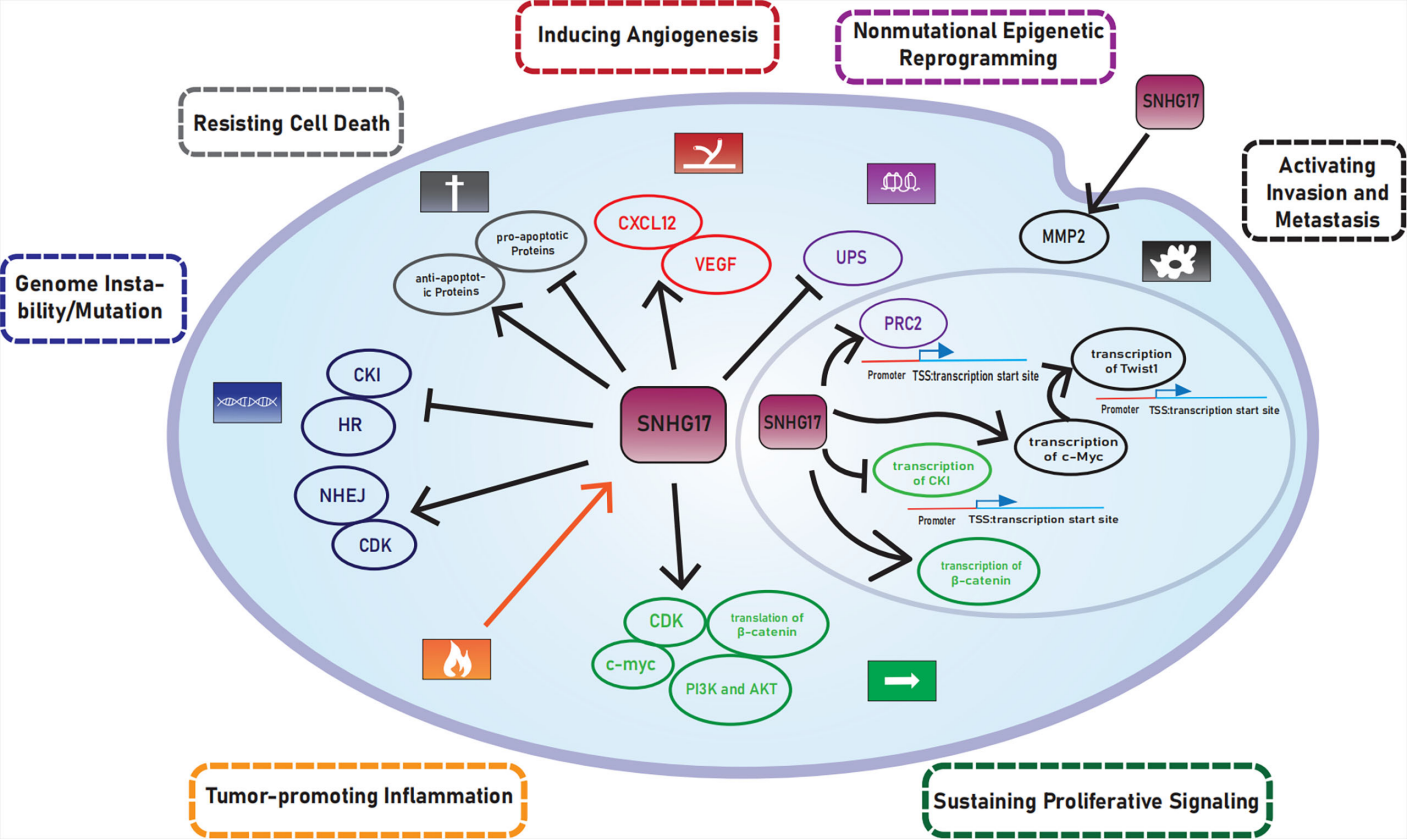
The relationship between SNHG17 and cancer hallmark.

SNHG17 was highly expressed in many cancers. Mechanistically, YY1 (33), TGF‐β1 (29), STAT3 (23, 28), and two positive feedback loops: SNHG17/miR-339-5p/STAT5A/SNHG17 (15), SNHG17/miR-339-5p/FOSL2/SNHG17 (26) are upstream regulators of SNHG17. This paper describes the mechanisms of SNHG17 that enable cells to gain cancer hallmarks (**Figure 5**) (**Table 2**). However, the relevance between SNHG17 and two other hallmarks, namely evading growth suppressors and polymorphic microbiomes, has been excluded due to the limitations of the published literature. Some proposed potential mechanisms of SNHG17 also require further experimental validation, which will be essential in future SNHG17 explorations. Meanwhile, SNHG17 regulation of its homolog snoRA71B through a positive feedback loop provides a new idea for the regulatory relationship between SNHG and snoRNA.

**Table 2:** The targets and mechanisms underlying the effects of SNHG17.

Cancer Types	Target/Regulatory Axis	Target Type	Action Mechanism	References
Cervical Cancer	SNHG17/miRNA-375-3p	miRNA	post-transcriptional regulation of genes as ceRNA	(42)
Pancreatic Carcinoma	SNHG17/miR-942	miRNA	post-transcriptional regulation of genes as ceRNA	(43)
Astrocytoma	SNHG17/miR-876-5p/ERLIN2	miRNA	post-transcriptional regulation of genes as ceRNA	(39)
Oral Squamous Cell Carcinoma	SNHG17/miR-384/ELF1/CTNNB1 SNHG17/miR-375/PAX6	miRNA	post-transcriptional regulation of genes as ceRNA	(34, 45)
Renal Cell Carcinoma	SNHG17/miR-328-3p/H2AX axis	miRNA	post-transcriptional regulation of genes as ceRNA	(46)
Esophageal Squamous Cell Carcinoma	SNHG17/miR-338-3p/SOX4	miRNA	post-transcriptional regulation of genes as ceRNA	(62)
Lung Adenocarcinoma	SNHG17/miR-193a-5p/NETO2 SNHG17/miR-485-5p/ WLS	miRNA	post-transcriptional regulation of genes as ceRNA	(30, 35)
Rectal Cancer	SNHG17/ miR-361-3p/STC2	miRNA	post-transcriptional regulation of genes as ceRNA	(36)
H. pylori-related Gastric Cancer	SNHG17/miR-3909/RING1/Rad51	miRNA	post-transcriptional regulation of genes as ceRNA	(94)
Colorectal Adenocarcinoma	SNHG17/miR-23a-3p/CXCL12	miRNA	post-transcriptional regulation of genes as ceRNA	(67)
Colorectal Cancer	SNHG17/miR-339-5p/FOSL2/SNHG17 positive feedback loop	miRNA	post-transcriptional regulation of genes as ceRNA	(26)
Ovarian Cancer	STAT3 /SNHG17/ miR-214-3p/CDK6	miRNA	post-transcriptional regulation of genes as ceRNA	(23)
Prostate Cancer	SNHG17/miR-339-5p/STAT5A/SNHG17 and SNORA71B	miRNA	post-transcriptional regulation of genes as ceRNA	(15)
Castration-Resistant Prostate Cancer	SNHG17/miR-144/CD51	miRNA	post-transcriptional regulation of genes as ceRNA	(64)
Hepatocellular Carcinoma	SNHG17 /miR-3180-3p/RFX1 SNHG17/LRPPRC/c-Myc	MiRNA protein	post-transcriptional regulation of genes as ceRNA reduce the ubiquitination and degradation	(63) (25)
Glioma	YY1/SNHG17/miR-506-3p/CTNNB1/Wnt/β-catenin	miRNA	post-transcriptional regulation of genes as ceRNA	(33)
Osteosarcoma	SNHG17 /miR-2861 /MMP2	miRNA	post-transcriptional regulation of genes as ceRNA	(47)
Colorectal Cancer	SNHG17/Trim23/PES1 SNHG17/EZH2/P57	protein	reduce the ubiquitination and degradation guide protein–RNA interaction as transcriptional co-repressor	(16, 26)
Gastric Cancer	SNHG17/EZH2/p15 and p57	protein	guide protein–RNA interaction as transcriptional co-repressor	(19)
Esophageal Squamous Cell Carcinoma	SNHG17/c-Jun/c-Myc	protein	guide protein–RNA interaction as transcriptional co-repressor	(29)

## Author contributions

Conceived the idea: NZ, MC. Wrote the majority of the manuscript: NZ. Collected the data, designed the tables and figures: NZ, YS, TW and XX. Critical revisions: MC and XX. All authors read and approved the final manuscript.

## Funding

This work was supported by Heilongjiang Postdoctoral Scientific Research Developmental Fund (LBH-Q20040).

## Acknowledgments

We are grateful for the help of Heilongjiang Human Resources and Social Security Bureau.

## Conflict of interest

The authors declare that the research was conducted in the absence of any commercial or financial relationships that could be construed as a potential conflict of interest.

## Publisher’s note

All claims expressed in this article are solely those of the authors and do not necessarily represent those of their affiliated organizations, or those of the publisher, the editors and the reviewers. Any product that may be evaluated in this article, or claim that may be made by its manufacturer, is not guaranteed or endorsed by the publisher.
