# Supplementary figures and images for: Enabling factor for cancer hallmark acquisition: Small nucleolar RNA host gene 17
Source: Front Oncol. 2022 Sep 14;12:974939. doi: 10.3389/fonc.2022.974939 (PMC9515549; doi:10.3389/fonc.2022.974939)

DEGs

IRG

1097

121

1672

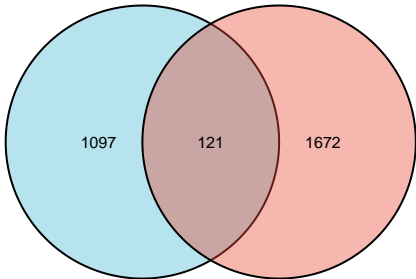

Supplement: Supplementary file 1 [file DataSheet_1.zip › A/╬1⁄4╢≈═╝_2022-04-11_15_34_51.pdf]

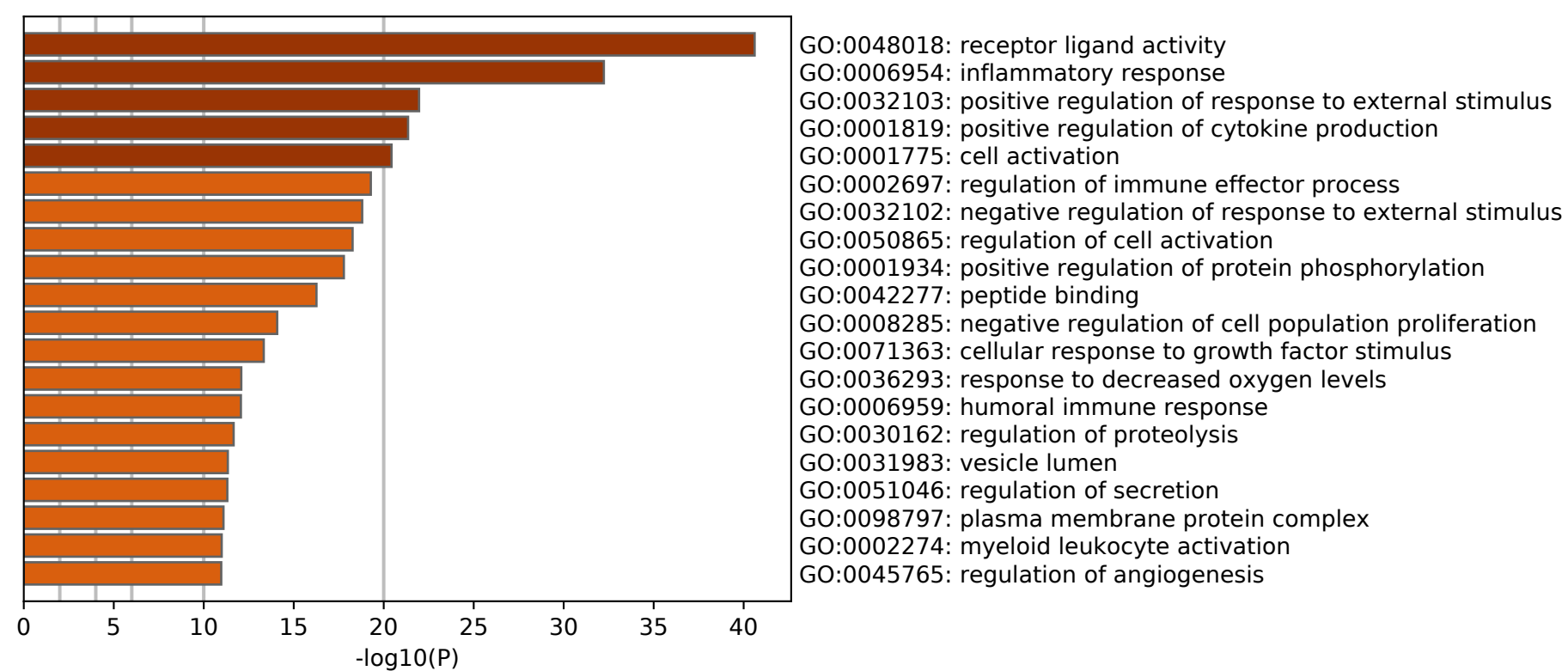

Supplement: Supplementary file 2 [file DataSheet_2.zip › B/metascape╬ñ╢≈═╝╜╗╝»GO╖╓╬÷.pdf]

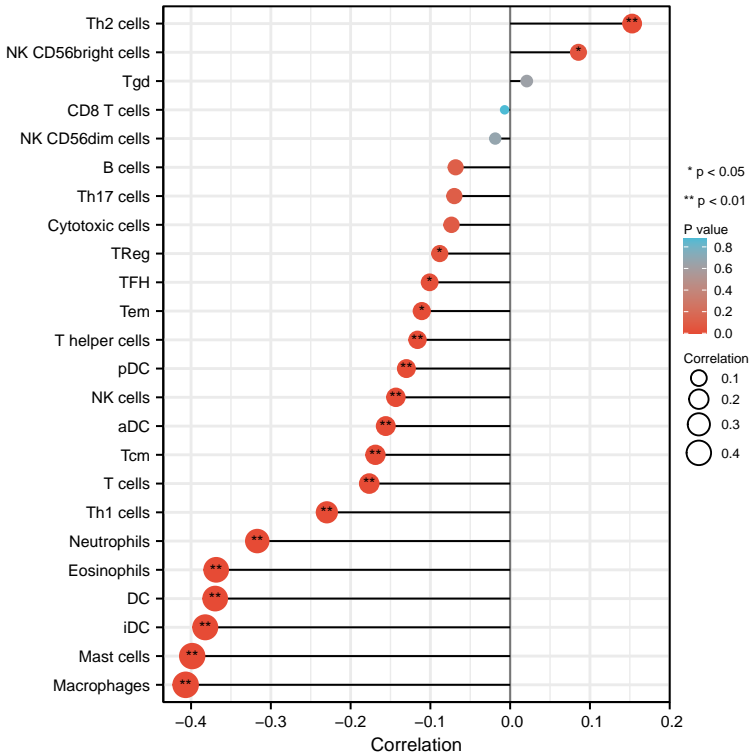

Supplement: Supplementary file 3 [file DataSheet_3.zip › SNHG17║═╕≈├Γ╥▀╧╕░√░⌠░⌠╠╟═╝_2022-04-27_16_28_47.pdf]

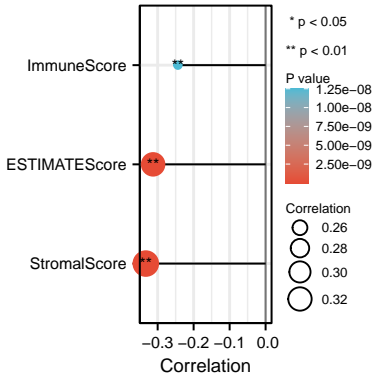

Supplement: Supplementary file 4 [file DataSheet_4.zip › ├Γ╥▀╞└╖╓░⌠░⌠╠╟═╝_2022-04-27_16_36_55.pdf]

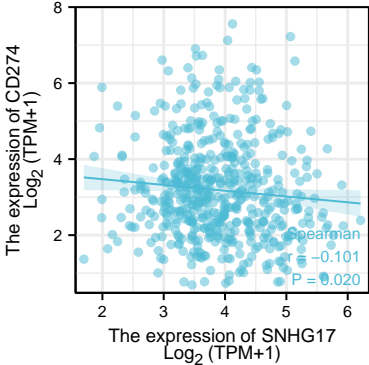

Supplement: Supplementary file 5 [file DataSheet_5.zip › ╖╓╫╙╧α╣╪╨╘╖╓╬÷_2022-06-13_20_02_37.pdf]
